# Meropenem Resistance among Acinetobacter Positive Clinical Samples in a Tertiary Care Centre in Nepal: A Descriptive Cross-sectional Study

**DOI:** 10.31729/jnma.5957

**Published:** 2021-09-30

**Authors:** Nisha Sharma, Bibechan Thapa, Ashirbad Acharya, Bijendra Raj Raghubanshi

**Affiliations:** 1 KIST Medical College and Teaching Hospital, Mahalaxmi-1, Lalitpur, Nepal; 2 Department of Microbiology, KIST Medical College and Teaching Hospital, Mahalaxmi-1, Lalitpur, Nepal

**Keywords:** *acinetobacter*, *anti-bacterial agents*, *carbapenem*, *drug resistance*, *meropenem*

## Abstract

**Introduction:**

Antimicrobial-resistant Acinetobacter species are implicated in a variety of infections including nosocomial bacteraemia, secondary meningitis and urinary tract infections. Carbapenem including meropenem-resistant Acinetobacter is recognized as one of the most difficult antimicrobial-resistant gram-negative bacilli to control and treat. It was classified as an urgent threat by Centers for Disease Control and Prevention in 2019 Antibiotic Resistance Threats Report. This study was carried out to determine the prevalence of meropenem resistance among acinetobacter positive clinical samples in a tertiary care centre.

**Methods:**

A descriptive cross-sectional study was carried out in microbiology department of Clinical Laboratory Services among Acinetobacter positive clinical samples of a tertiary care center in Nepal. The culture and sensitivity reports of various clinical samples from April 2018 to April 2020 which were positive for Acinetobacter species were taken from hospital records section. Convenience sampling was done. Meropenem-resistant Acinetobacter samples were studied. Ethical approval was received from Institutional Review Committee (Ref No. 076/77/40). Analysis of data was done using Statistical Package for the Social Sciences version 26. Point estimate at 95% Confidence Interval calculated with ferquency.

**Results:**

Out of 121 Acinetobacter isolates, prevalence of meropenem-resistant Acinetobacter was reported in 93 (76.9%) at 95% Confidence Interval (69.39-84.40). Among the meropenem-resistant Acinetobacter samples, most of the samples were collected from the sputum 70 (75.2%) followed by blood 8 (8.6%).

**Conclusions:**

High prevalence of meropenem-resistant Acinetobacter species in our hospital setting is alarming. In addition, there is emergence of resistance against even the last resort drugs which is creating a treatment crisis.

## INTRODUCTION

Acinetobacter species are aerobic Gram-negative, catalase-positive, oxidase-negative coccobacilli.^1^ They are associated with pneumonia, bacteraemia, urinary tract infections, meningitis and wound infections.^2^ It is intrinsically resistant to a broad range of antibiotics. There has been emergence of additional acquired resistance over last three decades.^3^

Carbapenems (imipenem, meropenem, or doripenem) have been considered as highly potent agents in the treatment of serious Acinetobacter infections.^4,5^ Meropenem Yearly Susceptibility Test Information Collection (MYSTIC) program that earlier revealed meropenem and imipenem as the most active agents against Acinetobacter baumannii over the years reported their marked increase in resistance rates.^6,7^ The emerging resistance has led to treatment failures and prolonged hospitalization.^8,9^

Centers for Diseases control and Prevention (CDC) has classified Carbapenem-resistant Acinetobacter as an urgent threat in 2019 Antibiotic Resistance Threats Report.^10^ This study was carried out to determine the prevalence of meropenem resistance among acinetobacter positive clinical samples in a tertiary care centre.

## METHODS

A descriptive cross-sectional study was carried out in the microbiology department of Clinical Laboratory Services (CLS) of KIST Medical College and Teaching Hospital (KISTMCTH), Lalitpur, which is a tertiary care center in Nepal. The culture and sensitivity reports of various clinical samples that were positive for Acinetobacter species between April 2018 and April 2020 were taken from the hospital records section. Ethical approval was received from Institutional Review Committee (Ref No. 076/77/40). Clinical samples [sputum, blood, urine, sample from endotracheal tube (ETT), tracheostomy foley's tip, tissue, pus, wound swab, bronchoalveolar lavage (BAL), high vaginal swab (HVS)] from which Acinetobacter species were isolated were included in this study. Also, the specimens growing more than one bacterial isolates along with Acinetobacter species were included. Acinetobacter samples that were resistant to meropenem were identified and analyzed. Those samples from which Acinetobacter species were not isolated and whose records did not reveal complete data were excluded.

Convenience sampling was done and the sample size (n) was calculated as,

n = Z^2^ × p × q / e^2^

  = (1.96)^2^ × 0.5 × (1 - 0.5) / (0.09)^2^

  = 119

Where,

n = required sample size
Z = 1.96 at 95% Confidence Interval (CI)
p = prevalence for maximum sample size, 50%
q = 1-p
e = margin of error, 9%

Our calculated sample size was 119 but our collected sample was 121.

The antimicrobial susceptibility of all isolates was determined by the standard Kirby Bauer disk diffusion method according to norms of the Clinical Laboratory Standards Institute (CSLI).^11^ Antibiotics included were Amikacin (30μg), Gentamicin (10μg), Trimethoprimsulfamethoxazole (1.25/23.75μg), Ceftriaxone (30μg), Ceftazidime (30μg), Ciprofloxacin (5μg), Piperacillin (30μg), Piperacillin/Tazobactam (100/10μg), meropenem (10μg), Imipenem (10μg),Tigecycline (15μg), Colistin (10μg) and Polymyxin B (300units) (Hi-Media India Pvt. Ltd). Findings were classified as sensitive, intermediate, and resistance.^11^

A bacterial isolate was considered non-susceptible to an antimicrobial agent when it tested resistant, intermediate or non-susceptible when using clinical breakpoints as interpretive criteria.^11^ Drug-resistant (DR) is non-susceptibility to one or more antibiotics which can be multidrug-resistant (MDR), extensively drug-resistant (XDR) or pandrug-resistant (PDR). MDR is defined as non-susceptibility to at least one agent in three or more antimicrobial categories. XDR is defined as non-susceptibility to at least one agent in all but two or fewer antimicrobial categories (i.e. bacterial isolates remain susceptible to only one or two categories). PDR is defined as non-susceptibility to all agents in all antimicrobial categories (i.e. no agents tested as susceptible for that organism).^12^

Data was entered and analysis was carried out through Statistical Package for the Social Sciences (SPSS) version 26. Descriptive analysis of all the data was done and point estimate at 95% Confidence Interval was calculated along with frequency and proportion for binary data.

## RESULTS

Report of various clinical samples from April 2018 to April 2020, were analyzed. There were 204 Acinetobacter isolated. Culture and sensitivity for meropenem was tested in 121 samples, prevalence of meropenem resistance was 93 (76.9%) (69.39-84.40 at 95% Confidence Interval).

Meropenem-resistant Acinetobacter was isolated slightly higher from males than females. Elderly patients of age above 45 were affected the most (Table 1).

**Table 1:** Table 1. Gender, age, and type of clinical sample distribution among meropenem-resistant Acinetobacter (n = 93).

Variables	n (%)
**Gender**	
Male	49 (52.7)
Female	44 (47.3)
**Age**	
<=15 years	2 (2.2)
16-45 years	30 (32.3)
>45 years	61 (65.5)

Among the meropenem-resistant Acinetobacter samples, most of the samples were collected from the sputum 70 (75.2%) followed by blood 8 (8.6%) (Table 2).

**Table 2:** Type of clinical samples distribution among meropenem-resistant Acinetobacter (n = 93).

Samples	n (%)
Sputum	70 (75.2)
Blood	8 (8.6)
Urine	2 (2.2)
Foley	1 (1.1)
ET tube	5 (5.3)
HVS	1 (1.1)
BAL	1 (1.1)
Trach	2 (2.2)
Wound	3 (3.2)
Total	93 (100)

Antimicrobial susceptibility of Acinetobacter positive clinical samples was tested for Amikacin, Ceftriaxone and other antibiotics which showed high resistancee along with meropenem (Table 3).

**Table 3:** Antimicrobial susceptibility of Acinetobacter positive clinical samples that were tested for meropenem (n = 21).

	Resistant n (%)	Sensitive n (%)	Intermediate n (%)	Total
Amikacin	114 (94.2)	5 (4.13)	2 (1.65)	121
Ampicillin	120 (99.2)	1 (0.83)	0 (0)	121
Cefotaxime	121 (100)	0 (0)	0 (0)	121
Ceftriaxone	120 (99.2)	1 (0.83)	0 (0)	121
Ciprofloxacin	116 (95.9)	5 (4.13)	0 (0)	121
Cotrimoxazole	107 (88.4)	11 (9.09)	3 (3.39)	121
Gentamicin	107 (88.4)	13 (10.7)	1 (1.13)	121
Meropenem	93 (76.9)	22 (18.2)	6 (7.81)	121

**Figure 1:**
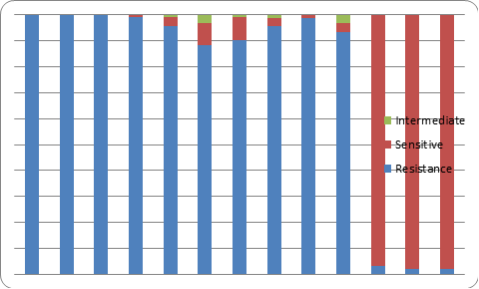
Antimicrobial susceptibility of Meropenem-resistant Acinetobacter to other antibiotics.

Meropenem resistant Acinetobacter were resistant to most of the first line antibiotics with 93 (100%) resistance towards cefotaxime, ampicillin and ceftriaxone (Figure 1).

They were mostly sensistive towards tigecycline, polymixin B and colistin. All meropenem-resistant isolates were multidrug-resistant, 77 (82.8%) isolates were extensively drug-resistant that was excluding one pandrug-resistant. Pandrug-resistant Acinetobacter isolate was reported from a sputum sample of a 78-year-old male.

Co-infection of Acinetobacter with other gram negative bacteria was observed in 14 cultures, Klebsiella was commonest among associates 11 (78.6 %) followed by Pesudomonas 2 (14.3 %) and Escherichia coli 1 (7.1%).

## DISCUSSION

Acinetobacter species are implicated in causing pneumonia, bacteraemia, urinary tract infections, meningitis and wound infections.^2^ It is intrinsically resistant to a broad range of antibiotics. However, there has been an emergence of additional acquired resistance over last three decades.^3^ Carbapenems (imipenem, meropenem, or doripenem) have been considered as extremely potent agents in the treatment of serious infections caused by Acinetobacter.^4-5^ There has been an increasing rise in carbapenem-resistant Acinetobacter baumannii (CRAB) one of the genetic factors being mainly the acquisition of carbapenem-hydrolysing oxacillinase genes.^3^

Carbapenem-resistant Acinetobacter was classified as an urgent threat in 2019 Antibiotic Resistance Threats Report by CDC.^10^ It is recognized as one of the most difficult antimicrobial-resistant gram-negative bacilli to control and treat. Reports reveal that the rapid increase in the prevalence of CRAB across the world has led to treatment failures and prolonged hospitalization.^8,9^ In 2017 the World Health Organization included CRAB in the global priority list of antibiotic-resistant bacteria as first priority (i.e. critical) to guide discovery, research and development of new drugs.^13^

Many studies have been carried out since decades to determine the emergence of carbapenem including meropenem-resistant Acinetobacter species. Meropenem Yearly Susceptibility Test Information Collection (MYSTIC) program studied the antimicrobial susceptibility of 490 Acinetobacter baumannii strains collected from 37 centers in 11 European countries from 1997 to 2000.^6^ It reported meropenem and imipenem as the most active agents against Acinetobacter baumannii, with resistance rates of 18% and 16%, respectively. However, the resistance rate was markedly increased to 43.4% for meropenem and 42.5% for imipenem as revealed by the subsequent data from 40 centers in 12 countries participating in the MYSTIC program (2006).^7^

In developing countries like Nepal too, resistance towards carbapenem, is increasing. In our study, prevalence of meropenem resistant Acinetobacter was observed in 93/121 (76.9%) samples which is very high compared to study done by Baral, et al. and Mishra, et al. where it was 28.5% and 50% respectively.^14-15^ However, higher prevalence was reported by Parajuli, et al. and Yadav, et al. where meropenem resistance was 84.4% and 89.4% respectively.^16-17^

More than half of meropenem-resistant Acinetobacter species along with Acinetobacter positive isolates were from respiratory specimen which was consistent with the finding that Acinetobacter are usually associated with lower respiratory tract infections.^18^ Highest number of infections were among patients of age group above 45 years which was similar to study done by Yadav, et.al. which also showed this bacteria to have a higher predilection for elderly patients.^17^

In our study resistance towards imipenem was 73.4% which was greater than twice reported by Raut, et al. in western Nepal.^19^ SENTRY study reported that Gram negative bacterial resistance to imipenem changed from 34.5% in 2006 to 59.8% in 2009 across the world.^20^

The increasing carbapenem-resistant Acinetobacter spp. has narrowed the treatment options with a combination of reserved drugs such as colistin, polymyxin B, and tigecycline being the only remaining choice to treat these cases.^21^

In multiple tertiary care centers in Nepal, Acinetobacter strains were reported to be 100% sensitive to colistin, polymixin B and tigecycline.^16,19,22^ However, in our study 2.3% of the total Acinetobacter isolates were resistant to each colistin and polymyxin-B and 3.8% were resistant to tigecycline. Meropenem resistant Acinetobacter were 100% resistant towards cefotaxime, ampicillin and ceftriaxone. All meropenem resistant Acinetobacter were multidrug-resistant, 77 (82.8%) isolates were extensively drug-resistant that was excluding one pandrug-resistant. There have been reports from across the world where resistance to colistin was highest in Asia followed by Europe. Reports from Bulgaria and Spain showed resistance rates 16.7% and 19.1%, respectively.^23^

Our study reported 6.9% Acinetobacter positive samples had co-infection with other gram negative bacteria, Klebsiella being the commonest (5.3%). These co-infections suggests the importance of choosing the appropriate antimicrobial therapy since it may increase patient mortality.^24^

This study has few limitations. To determine colistin susceptibility, it is recommended by International standard-setting organizations such as the Clinical and Laboratory Standards Institute (CLSI) and the European Committee on Antimicrobial Susceptibility Testing (EUCAST) joint polymyxin working group, March 2006, to use broth microdilution method, in which cation adjusted Mueller-Hinton broth is used.^25^ However, in our center, Kirby-Bauer Disc Diffusion Method is used. Also, this study was carried out in one tertiary care center. Hence, with the available data, only limited picture of resistant Acinetobacter species could be viewed.

## CONCLUSIONS

Prevalence of meropenem-resistant Acinetobacter species in our hospital settings is high and it is highly concerning. This has left reserve drugs like Colistin, Polymyxin B and Tigecycline as the only option available. However, there is development of resistance against these agents as well which signifies that there is an emerging treatment crisis to fight against drug-resistant Acinetobacter species. This can be minimized to some extent if antibiotics especially those with broad-spectrum activity and those identified as drugs of last resort, are used judiciously. Along with formulation of antimicrobial policies, solutions beyond the paradigm of antimicrobials should also be sought to address this problem.
